# Cyclodextrin Inhibits Caveolin‐1 to Regulate CD4^+^ T Cell Subset Differentiation and Ameliorate Rheumatoid Arthritis Progression

**DOI:** 10.1155/jimr/7245795

**Published:** 2026-08-12

**Authors:** Yajie Gao, Peiyao Zhang, Yinping Huo, Yongfu Wang, Lingyan Zhu

**Affiliations:** ^1^ The Central Lab, The First Affiliated Hospital of Baotou Medical College, Baotou, Inner Mongolia, China, btmc.cn; ^2^ Department of Infectious Diseases, Inner Mongolia People’s Hospital, Hohhot, Inner Mongolia, China, nmgyy.cn; ^3^ Department of Rheumatology and Immunology, The First Affiliated Hospital of Baotou Medical College, Baotou, Inner Mongolia, China, btmc.cn

**Keywords:** caveolin-1, cyclodextrin, rheumatoid arthritis, T cell, T cell differentiation

## Abstract

**Objective:**

Rheumatoid arthritis (RA) is a systemic autoimmune disease characterized by dynamically recurring inflammatory episodes, and targeting T cells has been shown to effectively alleviate RA. Caveolin‐1 (CAV‐1) is widely distributed in the membrane structures of immune cells and participates in the differentiation and homeostasis of CD4^+^ T cell subsets through signal transduction; however, its role in RA has not yet been investigated. This study aimed to examine the expression pattern and clinical relevance of CAV‐1 in RA, and to elucidate its regulatory role in CD4^+^ T cell‐mediated immune imbalance during disease progression.

**Methods:**

This retrospective clinical study was conducted to detect serum CAV‐1 levels in RA and healthy controls (HCs), characterize its expression in peripheral blood CD4^+^ T cells and synovial tissues, and evaluate its association with clinical parameters. A collagen‐induced arthritis (CIA) mouse model was established and treated with the CAV‐1 inhibitor hydroxypropyl‐β‐cyclodextrin (HP‐β‐CD). Joint pathology, CD4^+^ T cell infiltration, differentiation, and the expression of related inflammatory cytokines were assessed using histopathological and immunological approaches.

**Results:**

CAV‐1 expression in serum, CD4^+^ T cells, and synovial tissues was significantly higher in RA than in HC and was positively correlated with anti‐cyclic citrullinated peptide antibody (Anti‐CCP) and rheumatoid factor (RF) levels. HP‐β‐CD treatment markedly relieved joint inflammation and structural damage in CIA mice, reduced CD4^+^ T cell infiltration, and significantly suppressed the differentiation of T helper 1, T helper 17, and regulatory T (Treg) cells, accompanied by decreased expression of IFN‐γ, IL‐17A, and transforming growth factor‐β (TGF‐β).

**Conclusion:**

CAV‐1 is aberrantly upregulated in RA and contributes to disease pathogenesis by regulating CD4^+^ T cell differentiation. Targeting CAV‐1 may represent a promising strategy for RA diagnosis and immunomodulatory therapy.

## 1. Introduction

Caveolae are cave‐like lipid raft microdomains in which the spatial organization of membrane receptors regulates downstream signal transduction [1]. Caveolin‐1 (CAV‐1), a core structural protein of caveolae, is widely expressed in the membrane of various cell types, including immune cells [2, 3]. Through its caveolin scaffolding domain (CSD), CAV‐1 interacts with multiple signaling molecules and participates in the regulation of signal transduction and endocytosis, thereby influencing diverse cellular processes such as proliferation, differentiation, migration, apoptosis, and cytokine production [4, 5]. Increasing evidence has implicated CAV‐1 in immune regulation and disease progression in several autoimmune and inflammatory disorders, including multiple sclerosis [6], idiopathic pulmonary fibrosis [7], psoriasis [8], and chronic obstructive pulmonary disease (COPD) [9]. However, despite these observations, the expression characteristics and immunological role of CAV‐1 in rheumatoid arthritis (RA) have not yet been systematically investigated, and its potential pathological and clinical significance remain to be clarified.

RA is a chronic autoimmune disease characterized by persistent synovitis and synovial hyperplasia, accompanied by immune cell activation and increased vascular permeability, ultimately leading to progressive joint destruction and irreversible deformities [10]. Among immune cell populations, T cells are abundantly present in synovial tissues and synovial fluid and play a central role in the immunopathogenesis of RA [11, 12]. Immunometabolism studies have demonstrated that, during disease progression, T cells undergo extensive proliferation and differentiate into proinflammatory effector subsets [13], thereby activating immune‐related signaling pathways and amplifying inflammatory cascades that exacerbate synovial inflammation and joint damage. Consistent with these observations, therapeutic strategies targeting T cells have been shown to improve disease outcomes in RA [14], and suppression of Th17 cell differentiation effectively attenuates disease severity in the collagen‐induced arthritis (CIA) mouse model [15].

Although studies directly examining the involvement of CAV‐1 in T cells from patients with RA or CIA models are currently lacking, accumulating evidence indicates that CAV‐1 participates in the regulation of CD4^+^ T cell differentiation and immune homeostasis. Upregulation of CAV‐1 has been shown to suppress CD4^+^ T cell proliferation, differentiation into helper T (Th) cells, and cytokine production in murine asthma models [16], whereas CAV‐1 also promotes regulatory T (Treg) cell generation by enhancing transforming growth factor‐β (TGF‐β)–dependent T cell receptor signaling [17]. Given the increasing incidence of RA [18], the present study was designed to investigate the role of CAV‐1 in RA on CD4^+^ T cells and to elucidate its potential contribution to the disease pathogenesis.

## 2. Materials and Methods

### 2.1. Study Subjects

The clinical research of this study is a retrospective study. Patients with RA and healthy controls (HCs) who attended the Department of Rheumatology and Immunology or the Physical Examination Center of the First Affiliated Hospital of Baotou Medical College from 2024 to 2025 were enrolled in this study. The inclusion criteria for RA were (1) with a first‐time diagnosis of RA, meeting the 2010 American College of Rheumatology (ACR)/European League Against Rheumatism (EULAR) classification criteria [19]; (2) had not received nonsteroidal anti‐inflammatory drugs (NSAIDs), immunosuppressive agents, or other anti‐inflammatory medications prior to sample collection in the preceding 3 months; (3) with complete medical records; and (4) had a full understanding of the experimental principles and objectives and voluntarily agreed to participate in this study. The exclusion criteria were (1) having a history of systemic diseases, including hematological disorders, diabetes mellitus, malignant tumors, acute or chronic systemic infections, severe cardiopulmonary insufficiency, or severe hepatic or renal impairment; (2) coexisting autoimmune or rheumatic diseases, such as systemic lupus erythematosus, Sjögren’s syndrome, ankylosing spondylitis, and gout; (3) long‐term use of immunosuppressants and hormonal drugs; (4) pregnant or lactating women and patients with psychiatric disorders; and (5) with incomplete medical records or those unable to cooperate with clinical examinations.

Additionally, we collected and organized relevant data of subjects from the Smart System of Disease Management, including baseline information (age, gender, height, weight, and BMI), tender joint count (TJC), and swollen joint count (SJC) of RA patients, as well as laboratory test results. Disease activity of RA patients was assessed using the Disease Activity Score in 28 joints (DAS28) score, which was calculated based on the TJC28, SJC28, erythrocyte sedimentation rate (ESR), and general health (visual analog scale) (GH). The DAS28 was calculated using the following formula: DAS28 = 0.56 × √(TJC28) + 0.28 × √(SJC28) + 0.70 × ln(ESR) + 0.014 × GH.

We also collected synovial tissue from patients with osteoarthritis (OA) and RA. The inclusion criteria for OA were (1) meeting the ACR classification criteria for OA; (2) had not received NSAIDs, immunosuppressive agents, or other anti‐inflammatory medications prior to sample collection in the preceding 3 months; (3) with complete medical records; and (4) had a full understanding of the experimental principles and objectives and voluntarily agreed to participate in this study. The exclusion criteria were (1) having a history of systemic diseases, including hematological disorders, diabetes mellitus, malignant tumors, acute or chronic systemic infections, severe cardiopulmonary insufficiency, or severe hepatic or renal impairment; (2) coexisting autoimmune or rheumatic diseases, such as RA, ankylosing spondylitis, and systemic lupus erythematosus; (3) having a history of joint replacement surgery or intra‐articular corticosteroid injection within the past 6 months; (4) pregnant or lactating women, and patients with psychiatric disorders; and (5) with incomplete medical records or those unable to cooperate with clinical examinations. The inclusion and exclusion criteria for RA patients are the same as those mentioned above.

### 2.2. Isolation of CD4^+^ T Cells From Human Peripheral Blood

Peripheral blood samples were collected from RA patients and HC individuals into EDTA anticoagulant tubes and gently mixed. Peripheral blood mononuclear cells (PBMCs) were isolated using the Human Peripheral Blood Lymphocyte Separation Medium (LTS1077, TBD). Red blood cells were removed using Red Blood Cell Lysis Buffer (R1010, Solarbio). Subsequently, CD4^+^ T cells were isolated from PBMCs using the EasySep Human CD4^+^ T Cell Isolation Kit (17952, STEMCELL). Briefly, EasySep reagents were added to the cell suspension and incubated with magnetic particles following gentle mixing. The sample tubes were then placed in a magnetic separation rack, allowing magnetically labeled nontarget cells to adhere to the tube wall, while unlabeled CD4^+^ T cells remained in suspension. The supernatant was transferred to a new tube, yielding highly purified CD4^+^ T cells for subsequent experiments.

### 2.3. Measurement of Serum CAV‐1 Levels

Fasting peripheral venous blood samples were collected in the morning from enrolled participants (HC, *n* = 40; RA, *n* = 40). After standing at room temperature for 2 h, the samples were centrifuged at 3000 rpm for 10 min to separate the serum. The serum was aliquoted into 1.5 mL microcentrifuge tubes and stored at −80°C. Serum levels of CAV‐1 were measured using a Human Caveolin‐1 ELISA Kit (BY‐EH110811, BY Abscience), and correlation analyses were performed to evaluate the associations between serum CAV‐1 levels and laboratory parameters in patients with RA. Moreover, we drew receiver operating characteristic (ROC) curves to assess the diagnostic effect of a certain index on the tester, calculate the area under the ROC curve (AUC), and determine the optimal threshold based on the Youden index.

### 2.4. Experimental Animals

We purchased specific‐pathogen‐free grade DBA/1 male mice (6–7 weeks old; 18–22 g) from GemPharmatech Co., Ltd. (Nanjing, China). The mice were fed and housed in an environment with ~50%–60% humidity and at temperatures between 20°C and 26°C on a 12 h light/dark cycle, with free access to food and water. After a 1‐week acclimatization period, the experiments were commenced. All animal studies were conducted in accordance with the U.S. National Research Council Guidelines for the Care and Use of Laboratory Animals.

### 2.5. Establishment of CIA Mouse Model

Bovine type II collagen (20021, Chondrex) was emulsified with complete Freund’s adjuvant (4 mg/mL; 7001, Chondrex) at a 1:1 ratio using a 2 mL screw‐cap syringe. The prepared emulsion was injected subcutaneously at a site ~2 cm from the base of the tail of DBA/1 mice. A booster immunization was administered on day 21 using the same procedure to establish the CIA model. One week after immunization, CIA mice were randomly divided into three groups: control, CIA, and CIA + HP‑β‑CD (*n* = 6 per group). Hydroxypropyl‐β‐cyclodextrin (HP‐β‐CD) (778966‑100G, MERCK, dissolved in saline) was administered via intraperitoneal injection every other day to CIA mice (4 g/kg) to achieve inhibition of CAV‑1. The control and CIA groups received equal doses of saline. 4 weeks later, mice were anesthetized, and micro‐computed tomography (micro‐CT) images of the hind limbs were acquired using a high‐resolution in vivo X‐ray cone‐beam scanner to evaluate joint structural changes. Mice were then euthanized, and hind limbs were collected for histological evaluation using hematoxylin and eosin (HE) staining and toluidine blue (TB) staining. In addition, spleen and lymph node tissues were harvested for immunofluorescence (IF) and immunohistochemical (IHC) analysis.

### 2.6. Histopathological Analysis

Histopathological evaluation was performed using HE staining and TB staining [20]. HE‐stained sections were assessed for the severity of inflammation, synovial hyperplasia, cartilage destruction, and bone erosion using a semiquantitative scoring system ranging from 0 to 5, with higher scores indicating greater severity (0, normal; 5, severe damage with extensive infiltration or tissue loss). TB staining was used to evaluate the extent of chondrocyte loss and/or cartilage matrix degradation, which was also scored on a scale of 0–5, with higher scores reflecting more severe cartilage damage (0, normal; 5, severe damage with multifocal severe chondrocyte loss and/or collagen disruption). Each slide was scored independently by two observers, and the mean values were used for subsequent analysis.

### 2.7. IF and Immunohistochemistry Staining

IF and IHC staining were performed on cell samples and tissue sections following standard procedures. Briefly, samples were subjected to antigen retrieval, followed by blocking with an appropriate blocking solution. The samples were then incubated with primary antibodies, followed by incubation with the corresponding secondary antibodies. Signals were visualized using fluorescence or chromogenic detection and captured the images. Quantitative analysis of fluorescence intensity and optical density was performed using Image J and Image‐Pro Plus (performed by Wuhan Sevier Biotechnology Co., Ltd.).

For IF staining, human peripheral blood CD4^+^ T cells isolated as described in Section 2.1, synovial tissue sections from patients with OA and RA, and spleen tissues from control, CIA, and CIA+HP‐β‐CD mice were stained with anti‐CD4 (1:400, 19068‐1‐AP, Proteintech) and anti‐CAV‐1 (1:100, CL488‐66067, Proteintech). Lymph node tissues from control, CIA, and CIA+HP‐β‐CD mice were stained with anti‐CD4 (1:800, 67786‐1‐Ig, Proteintech), anti‐CXCR3 (1:50, 26756‐1‐AP, Proteintech), anti‐CCR6 (1:400, 66801‐1‐Ig, Proteintech), and anti‐FOXP3 (1:1000, GB112325, Servicebio). For IHC staining, lymph node sections from control, CIA, and CIA+HP‐β‐CD mice were stained with anti–IFN‐γ (1:200, bs‐0480R, Bioss), anti–IL‐17A (1:500, GB11110‐1, Servicebio), and anti–TGF‐β (1:500, GB11179, Servicebio).

### 2.8. Statistical Analysis

All statistical analyses were performed using GraphPad Prism (10.0). Continuous variables with a normal distribution are presented as mean ± standard error of the mean (SEM), whereas variables with a non‐normal distribution are expressed as the median (P25 and P75). Comparisons between two groups were conducted using the independent‐samples *t*‐test or the Mann–Whitney *U* test, as appropriate. Comparisons among more than two groups were performed using one‐way ANOVA or the Kruskal–Wallis test. Categorical data were analyzed using the Fisher’s exact test. For correlation analyses, Pearson’s correlation coefficient was applied to normally distributed data, whereas Spearman’s correlation coefficient was used for non‐normally distributed or ordinal variables. *p* < 0.05 was considered statistically significant.

### 2.9. Ethics Statement

The acquisition of clinical samples for this study was approved by the Ethics Committee of the First Affiliated Hospital of Baotou Medical College (Approval Number K060‐01) and was conducted in accordance with the Declaration of Helsinki (1964). The participants provided their written informed consent to participate in this study. The animal study was approved by the Animal Care and Use Committee of the First Affiliated Hospital of Baotou Medical College and conducted in accordance with the Guide for the Care and Use of Laboratory Animals (Approval Number D024). All the studies were conducted in accordance with the local legislation and institutional requirements.

## 3. Results

### 3.1. CAV‐1 Is Upregulated in RA and Exhibits Diagnostic Potential

**Figure 1:**
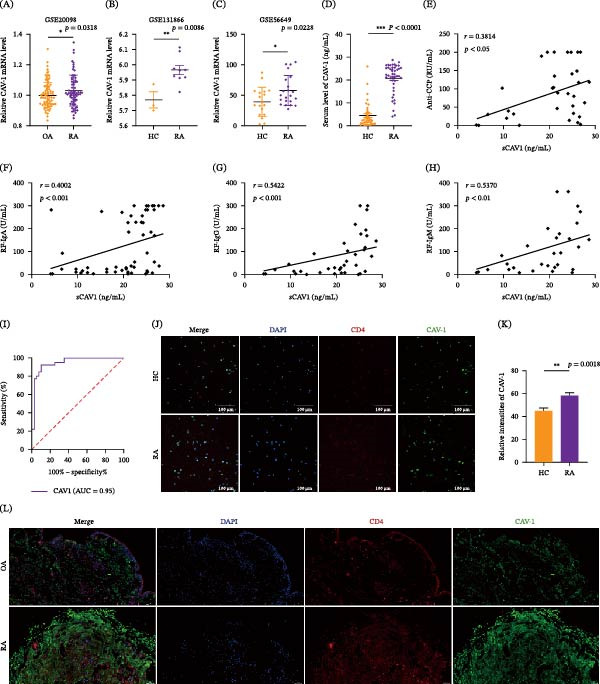
Upregulation of CAV‐1 expression in patients with RA. (A–C) CAV‐1 expression in CD4^+^ T cells from patients with RA based on transcriptomic datasets GSE20098 (OA *n* = 101 and RA *n* = 72), GSE131866 (HC *n* = 3 and RA *n* = 9), and GSE56649 (HC *n* = 18 and RA *n* = 22). (D) Serum levels of CAV‐1 (HC *n* = 40 and RA *n* = 40). (E–H) Correlations between serum CAV‐1 levels and Anti‐CCP, RF‐IgA, RF‐IgG, and RF‐IgM in patients with RA. (I) ROC curve analysis evaluating the diagnostic performance of serum CAV‐1 in RA. (J) Representative IF staining of CD4 and CAV‐1 in peripheral blood CD4^+^ T cells (HC *n* = 3 and RA *n* = 3). (K) Quantitative analysis of CAV‐1 fluorescence intensity in CD4^+^ T cells. (L) Representative IF staining of CD4 and CAV‐1 in synovial tissues (OA *n* = 3 and RA *n* = 3). All data are presented as mean ± SEM from at least three independent experiments. Statistical significance was determined using Student’s *t*‐test.  ^∗^ *p* < 0.05,  ^∗∗^ *p* < 0.01,  ^∗∗∗^ *p* < 0.001 versus the HC or OA group.

Given the reported dysregulation of CAV‐1 in autoimmune diseases, we investigated its expression and clinical relevance in RA. Analysis of CD4^+^ T cell transcriptomic datasets from the GEO database (GSE20098, GSE131866, and GSE56649) revealed a significant upregulation of CAV‐1 expression in RA (Figure 1A–C).

Based on the inclusion and exclusion criteria, a total of 40 HC and 40 RA samples were enrolled in this study. Baseline characteristics (age, gender, height, weight, and BMI) were compared between the two groups. No statistically significant differences were observed in any baseline variables except height. Given that BMI, a comprehensive indicator reflecting body composition, did not differ significantly between groups, the observed difference in height was considered unlikely to influence participant enrollment or the interpretation of study outcomes. In addition, laboratory parameters of RA patients were collected and analyzed, including ESR, C‐reactive protein (CRP), anti‐cyclic citrullinated peptide antibody (Anti‐CCP), mutated citrullinated vimentin antibody (MCV), immunoglobulin M‐rheumatoid factor (IgM‐RF), IgA‐RF, IgG‐RF, IgG, IgA, and IgM. The disease activity was assessed using the DAS28 score. Detailed demographic and clinical characteristics of the participants are summarized in Table 1. The results demonstrated that serum CAV‐1 levels were significantly elevated in RA (Figure 1D). Correlation analyses between serum CAV‐1 and clinical laboratory parameters revealed significant positive associations with Anti‐CCP, RF‐IgA, RF‐IgG, and RF‐IgM (Figure 1E–H). ROC curve analysis demonstrated a high diagnostic accuracy of serum CAV‐1 for RA, with an AUC of 0.95, an optimal cutoff value of 9.6945 ng/mL, a sensitivity of 97.56%, and a specificity of 94.74% (Figure 1I). These results indicate that serum CAV‐1 exhibits high diagnostic accuracy for RA and may serve as a potential biomarker for the disease diagnosis.

**Table 1:** Baseline characteristics of HC and RA.

Variables	HC	RA	*p*‐Value
Male/female (*n*)	20/20	17/23	ns.
Age (years)	55.23 ± 11.67	58.89 ± 15.01	ns.
Height (cm)	168.50 ± 1.30	164.00 ± 1.13	<0.05
Weight (kg)	62.60 ± 1.44	58.00 (53.50, 65.00)	ns.
BMI (kg/m^2^)	21.93 ± 0.24	22.58 ± 0.53	ns.
DAS28	—	5.22 ± 1.32	—
ESR (mm/H)	—	29.00 (19.50, 51.00)	—
CRP (mg/L)	—	13.60 (8.35, 25.10)	—
Anti‐CCP (RU/mL)	—	102.00 (31.00, 200.00)	—
MCV (RU/mL)	—	1000.00 (215.00, 1000.00)	—
IgM‐RF (U/mL)	—	125.00 (28.90, 363.00)	—
IgA‐RF (U/mL)	—	94.00 (9.00, 300.00)	—
IgG‐RF (U/mL)	—	42.00 (10.00, 147.00)	—
IgG (g/L)	—	15.15 ± 4.45	—
IgA (g/L)	—	3.44 ± 1.13	—
IgM (g/L)	—	1.38 ± 0.49	—

*Note:* Data are expressed as mean ± SEM or median (P25 and P75). Anti‐CCP, anti‐cyclic citrullinated peptide antibody; MCV, mutated citrullinated vimentin antibody.

Abbreviations: BMI, body mass index; CRP, C‐reactive protein; DAS28, disease activity score 28; ESR, erythrocyte sedimentation rate; RF, rheumatoid factor.

Consistent with the serum findings, CAV‐1 expression levels in both peripheral blood CD4^+^ T cells and synovial tissues were markedly increased in RA compared with HC (Figure 1J–L). Collectively, these results suggest that aberrant upregulation of CAV‐1 is closely associated with RA pathogenesis and provide a rationale for further investigation into the regulatory role of CAV‐1 in the disease progression.

### 3.2. HP‐β‐CD Attenuates Disease Severity and Reduces CAV‐1 Expression in CIA Mice

**Figure 2:**
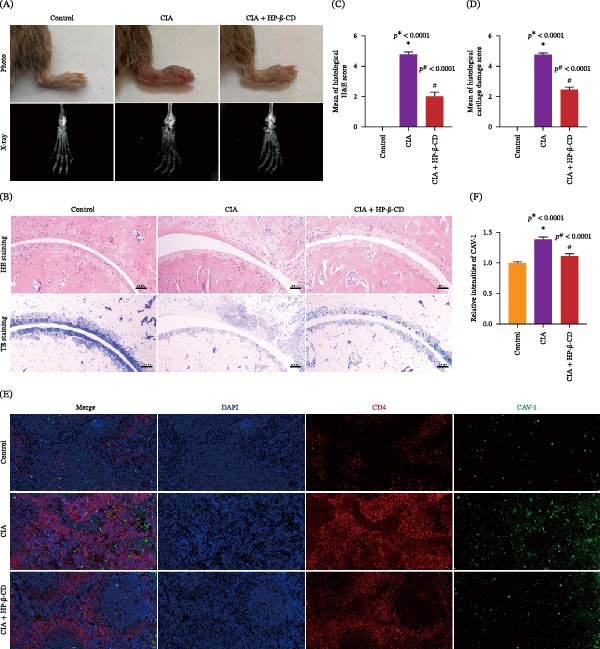
The CAV‐1 inhibitor HP‐β‐CD attenuated disease progression in CIA mice. (A) Representative images of hind limbs and corresponding radiographic (micro‐CT) evaluation of joint changes in mice. (B) Representative HE and TB staining of joint sections. (C) Histopathological scores based on HE staining. (D) Joint damage scores. (E) Representative IF staining of CD4 and CAV‐1 in spleen tissues from each group. (F) Quantitative analysis of CAV‐1 fluorescence intensity in spleen tissues. Results are representative of three independent biological replicates. All data are presented as mean ± SEM (control *n* = 6, CIA *n* = 6 and CIA + HP‐β‐CD *n* = 6). Statistical significance was determined using one‐way ANOVA.  ^∗^ *p* < 0.05 versus the control group; ^#^ *p* < 0.01 versus the CIA group.

To further explore the functional role of CAV‐1 in RA, the CIA mouse model was treated with HP‐β‐CD, which was used to disrupt caveolae and inhibit CAV‐1‐dependent signaling [21–23]. HP‐β‐CD treatment significantly relieved joint inflammation and swelling, reduced joint destruction, and improved the overall joint architecture compared with CIA mice (Figure 2A–D). Histological analysis revealed decreased inflammatory cell infiltration and attenuated cartilage damage following HP‐β‐CD administration.

Consistently, the expression and distribution of CD4^+^CAV‐1^+^ T cells in splenic tissues were markedly increased in CIA mice compared with controls, whereas HP‐β‐CD treatment significantly reduced both CD4 and CAV‐1 expression levels (Figure 2E, F). These findings indicate that CAV‐1 inhibition partially mitigates the disease progression in CIA mice.

### 3.3. HP‐β‐CD Suppresses CD4^+^ T Cell Subset Differentiation in CIA Mice

**Figure 3:**
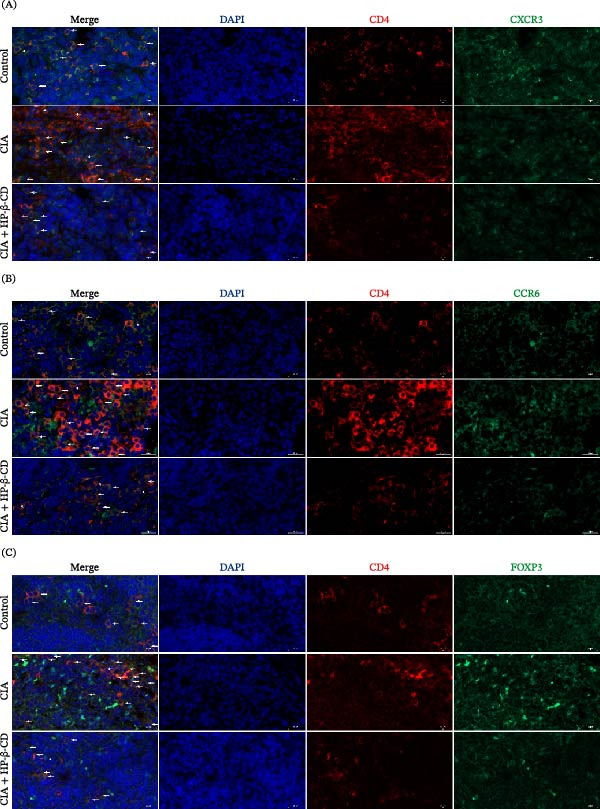
HP‐β‐CD suppressed differentiation of Th1, Th17, and Treg cells. Differentiation of Th1 (A), Th17 (B), and Treg (C) cells in lymph nodes of mice. Results are representative of three independent biological replicates (control *n* = 6, CIA *n* = 6 and CIA + HP‐β‐CD *n* = 6).

**Figure 4:**
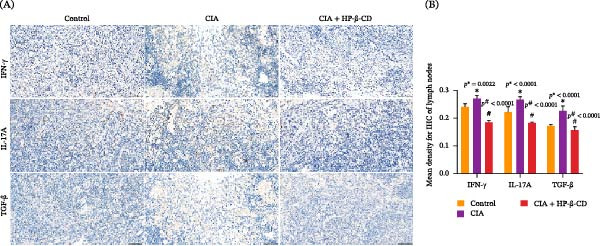
HP‐β‐CD reduced the expression of IFN‐γ, IL‐17A, and TGF‐β. (A) Representative IHC staining of IFN‐γ, IL‐17A, and TGF‐β in lymph nodes of mice. (B) Quantitative analysis of IHC staining expressed as mean optical density. Results are representative of three independent biological replicates. All data are presented as mean ± SEM (control *n* = 6, CIA *n* = 6 and CIA + HP‐β‐CD *n* = 6). Statistical significance was determined using one‐way ANOVA.  ^∗^ *p* < 0.05 versus the control group; ^#^ *p* < 0.01 versus the CIA group.

Transcriptomic analysis of CD4^+^ T cells from non‐RA and RA individuals by Pratt et al. [24] demonstrated significant enrichment of Th cell differentiation‐related pathways by gene set enrichment analysis, supporting that CD4^+^ T cells and their differentiation are pivotal in RA progression. Based on this, we examined whether CAV‐1 inhibition affected T cell subset differentiation. In CIA mice, the proportions of Th1, Th17, and Treg cells in lymph nodes were significantly increased compared with controls (Figure 3A–C), accompanied by elevated levels of their signature cytokines IFN‐γ, IL‐17A, and TGF‐β (Figure 4A, B). Notably, HP‐β‐CD treatment markedly reduced the differentiation of these CD4^+^ T cell subsets and suppressed the production of associated cytokines.

Together, these results demonstrate that CAV‐1 is upregulated in RA and contributes to disease progression moderately, at least in part, by promoting CD4^+^ T cell activation and differentiation, and that inhibition of CAV‐1 ameliorates inflammatory arthritis by modulating pathogenic T cell responses.

## 4. Discussion

The focus of this study was to investigate the mechanistic role of CAV‐1 in RA. As a ubiquitously expressed membrane‐associated scaffolding protein, CAV‐1 has been implicated in the pathogenesis of multiple autoimmune disorders. However, its immunomodulatory function contribution to RA remains largely unexplored. In synovial fluid‐derived fibroblast‐like synoviocytes, CAV‐1 mediates the expression of C–C chemokine ligand 2/monocyte chemoattractant protein‐1, which then promotes the development of RA synovitis through the production of pro‐inflammatory cytokines and other mediators [25]. Besides, the function of CAV‐1 has not been unequivocally determined, more studies are needed to confirm the role of CAV‐1 in inflammatory processes related to RA.

Our findings demonstrated that CAV‐1 expression was markedly elevated in both patients with RA and CIA mice, suggesting a potential involvement of CAV‐1 in the pathogenesis of inflammatory arthritis. Furthermore, serum CAV‐1 levels were positively correlated with established clinical diagnostic markers of RA. These associations indicated that increased CAV‐1 expression was closely linked to disease‐related immunological abnormalities. Moreover, CAV‐1 may serve as a promising biomarker for RA diagnosis, combining CAV‐1 with currently established diagnostic indicators may further improve diagnostic sensitivity and accuracy, thereby facilitating earlier disease detection and timely therapeutic intervention. Nevertheless, larger multicenter studies are warranted to validate its clinical utility and diagnostic value in diverse patient populations.

Our study indicated that HP‐β‐CD‐mediated inhibition of CAV‐1 partially suppressed CD4^+^ T cell differentiation, reduced the severity of joint pathological damage, and attenuated systemic inflammation in CIA mice. Th and Treg cells represent central CD4^+^ T cell subsets that govern immune tolerance, homeostasis, and pathogenic immune responses [26]. In RA, dysregulated inflammation skews T cell differentiation toward proinflammatory Th1 and Th17 lineages while impairing immunosuppressive Treg function, thereby sustaining chronic synovial inflammation and joint destruction [27–30]. Consistent with this paradigm, our study demonstrates that CAV‐1 expression is observably increased in CIA mice, accompanied by enhanced Th1 and Th17 differentiation and excessive cytokine production. However, HP‐β‐CD significantly alleviated joint pathology in CIA mice, reduced CD4^+^ T cell accumulation, and attenuated inflammatory progression. Taken together, our results highlight CAV‐1 as a previously underappreciated regulator of RA pathogenesis, providing new insights into its role in the disease progression. Beyond suppressing proinflammatory Th1 and Th17 responses, HP‐β‐CD treatment also reduced the proportion of Treg cells and TGF‐β expression in CIA mice. Although Treg cells are generally regarded as protective in autoimmunity, their expansion in chronic inflammatory settings may reflect a compensatory but functionally insufficient response.

TGF‐β is a pivotal determinant of CD4^+^ T cell fate, recent single‐cell RNA sequencing studies have revealed that TGF‐β regulates T cell heterogeneity, and is capable of promoting either Treg or Th17 differentiation depending on the cytokine milieu [31, 32]. In the presence of IL‐6 or IL‐21, TGF‐β drives Th17 differentiation through STAT3 and RORγt activation, whereas TGF‐β signaling alone induces Foxp3 expression and favors Treg lineage commitment [33, 34]. Our findings suggest that dysregulated TGF‐β signaling contributes to an imbalanced Th17/Treg axis during RA progression and that CAV‐1 inhibition could restore immune equilibrium to some extent.

Mechanistically, CAV‐1 functions as a key organizer of caveolae‐mediated membrane compartmentalization and signaling. The CSD of CAV‐1 dynamically interacts with multiple signaling proteins, including TGF‐β receptors, thereby regulating receptor localization, internalization, and downstream Smad activation [35]. TGF‐β receptors can be internalized via clathrin‐dependent pathways that promote Smad signaling or caveolae‐dependent pathways that facilitate receptor degradation [36]. CAV‐1 enhances the recruitment of TGF‐β receptors into caveolae, suppressing Smad2/3 phosphorylation, and attenuating TGF‐β transcriptional activity [37–41]. Consistent with this regulatory role, inhibition of CAV‐1 by HP‐β‐CD likely disrupts caveolae‐dependent receptor trafficking, thereby reshaping TGF‐β signaling output and ultimately modulating CD4^+^ T cell differentiation in RA.

Notably, accumulating evidence also indicates that CAV‐1 and TGF‐β signaling engage in bidirectional regulation. TGF‐β has been reported to downregulate CAV‐1 expression in a dose‐dependent manner, suggesting the existence of a dynamic feedback loop that fine‐tunes immune responses [42]. Our study provides functional evidence that targeting CAV‐1 may alter TGF‐β–associated immune regulation and mitigate RA disease severity; however, the precise molecular interactions between CAV‐1 phosphorylation [43], caveolae dynamics [1], and TGF‐β receptor trafficking [44, 45] remain to be fully elucidated. Future studies focusing on the CAV‐1/TGF‐β signaling axis will be essential to clarify its role in T cell fate decisions and to assess its therapeutic potential in RA.

## 5. Conclusion

This study demonstrates that CAV‐1 is significantly upregulated in patients with RA and is closely associated with disease‐related clinical parameters. Pharmacological inhibition of CAV‐1 partially alleviates joint inflammation and tissue damage in CIA mice by suppressing CD4^+^ T cell infiltration and dysregulated T cell subset differentiation. These findings suggest that CAV‐1 is highly likely to be a key immunoregulatory factor in RA and thus a potential diagnostic and therapeutic target.

## Author Contributions

Yajie Gao and Peiyao Zhang contributed to study design, conducted the experimental work, data analysis, and manuscript drafting. Yinping Huo participated in data acquisition and analysis. Yongfu Wang and Lingyan Zhu were responsible for funding acquisition and manuscript review.

## Funding

This work was supported by the National Natural Science Foundation of China (Grant 82160309), the Natural Science Foundation of Inner Mongolia (Grants 2025QN08069 and 2025LHMS08006), the Science and Technology Program of the Joint Fund of Scientific Research for the Public Hospitals of Inner Mongolia Academy of Medical Sciences (Grant 2024GLLH0527), and the Young and Middle‐aged Scientific and Technological Backbone Support Program of Baotou Medical College (Grant BYJJ‐QNGG202401).

## Conflicts of Interest

The authors declare no conflicts of interest.

## Data Availability

The supporting data for this study are available upon request from the authors.
